# The Effect of Working Memory Updating Ability on Spatial Insight Problem Solving: Evidence From Behavior and Eye Movement Studies

**DOI:** 10.3389/fpsyg.2019.00927

**Published:** 2019-04-24

**Authors:** Qiang Xing, Zheyi Lu, Jing Hu

**Affiliations:** ^1^ Department of Psychology, School of Education, Guangzhou University, Guangzhou, China; ^2^ Guangzhou Sport University, Guangzhou, China

**Keywords:** working memory updating, problem solving, insight, representation restructuring, eye movement

## Abstract

It still remains uncertain whether working memory updating ability influences spatial insight problem solving and whether working memory updating ability plays a role in the representation restructuring phase. The current study explored the correlation of working memory updating ability and spatial insight problem solving by behavior and eye movement experiments, and the results showed that high working memory updating ability individuals spend significant shorter time to solve spatial insight problem than low working memory updating ability individuals. For participants with high or low working memory updating ability, the underlying mechanism of spatial insight problem solving is sudden rather than incremental, which demonstrated that the working memory updating ability did not influence the representation restructuring phase. Working memory updating ability influences spatial problem solving, and it works critically in the problem space search phase, while the restructuring phase is sudden and immediate, which is not influenced by working memory updating ability. The representation restructuring tends to be spontaneous.

## Introduction

Creative thinking plays an important role in human society, facilitating individual and social development. Creative thinking is ubiquitous in current society, production, transportation, and entertainment, especially in the field of education. Researchers attempt to identify factors that influence creative thinking (Chirico et al., 2018) and ways to improve creative thinking (Nurdyani et al., 2018). Insight is an important cognitive process in creative thinking. Bowden and Jung-Beeman (2007) argued that insight is a phenomenon accompanied by an “aha” experience in which participants suddenly and intuitively understand complex perceptual situations or capture the intrinsic property of things.

Ash and Wiley (2006) indicated that the process of insight problem solving consists of three main phases: the initial representation phase, the faulty problem space search phase, and the post-impasse problem representation restructuring phase. In the initial representation phase, the problem solver would inappropriately represent the problem. The faulty problem space search phase may lead to the form of impasse. In post-impasse problem representation restructuring phase, the solver would overcome the impasse and restructure the problem representation and then get the problem successfully solved. The non-insight problem (analytical problem) can be solved through the search for problem representation, but the solution of insight problem needs to be restructured into an appropriate representation after the faulty problem space searching, only in this way can the insight problem be solved. Fleck and Weisberg (2004) hold the view that restructuring occurs in a few small, incremental, reportable steps that change the initial representation after a problem-solving failure, during which consciousness and cognitive resources should be involved. By contrast, some studies proposed that restructuring contains subconscious changes in the representation of problems, which is an unreportable and sudden process. It is tended to be spontaneous (Jung-Beeman et al., 2004; Öllinger et al., 2006). Overall, problem representation restructuring is essential to cognitive mechanism underlying insight problem solving, but whether the consciousness involved in representation restructuring phase remains debate.

In the process of insight problem solving, executive function, emotional state, time pressure, expected reward, and embodied guidance (Xing et al., 2018) would have an impact on it. Gilhooly and Fioratou (2009) investigated the individual differences in executive function in insight and non-insight problem solving and found that the achievement of insight problem solving is only related to the working memory subcomponent of executive function, and the span of working memory could well predict the achievement of insight and non-insight problem solving. On the other hand, the insight and non-insight problem solving are irrelevant with the inhibition and transformation, that is, the process of insight problem solving does not seem to need the participation of inhibition and transformation. Executive functions are generally considered to include three sub-components: responses inhibition, transformation of mental sets, and working memory updating (WMU; Huizinga et al., 2006; Zhou, 2013). In addition, WMU ability is closely correlated to fluid intelligence and advanced cognitive abilities (Chen and Li, 2007; Engle, 2010). Chein and Weisberg (2014) compared the differences of individuals with different working memory capacities and attentional abilities in solving insight problem and found that individuals’ differences in working memory capacity and attention ability can significantly predict the score of insight problem solving. The process of insight problem solving required working memory and attention resources, and the updating ability is used to maintain multiple representation and quickly update the representation in a short-term information storage and processing system when the specific stimuli occur. WMU ability shows the individual’s working memory capacity and information updating ability and mainly involves the cognitive resources and conscious process (Collette and Linden, 2002; Zhou, 2013). Furthermore, quantities of studies have found that WMU ability has a significant predictive effect on insight problem solving (Jung-Beeman et al., 2004; Chein and Weisberg, 2014; Xing et al., 2017).

Verbal insight problem has been proved to be correlated with executive function, and the results suggested that executive function influences searching within the problem space but not the problem representation restructuring phase (Xing et al., 2017). The two key processes in representation restructuring are constraint relaxation and chunk decomposition (Luo and Niki, 2003; Luo et al., 2004; Zhang et al., 2019). In this case, the representation restructuring phase in spatial insight problem is similar to that in verbal insight problem. Classic spatial insight problem includes the nine-dot problem, the mathematic arithmetic problem, and tumor-laser radiation problem. The nine-dot problem requires problem solvers to draw four connected straight lines to connect all of nine dots, and the pen used for drawing is not allowed to be lifted from the paper (Chein et al., 2010). Chein et al. (2010) found that higher spatial working memory capacity was related to faster solution of nine-dot problem. The matchstick arithmetic problem requires problem solvers to move one or more matchstick to ensure the equation make sense (Öllinger et al., 2006). Recent study proposed that tight chunk in matchstick arithmetic problem is more difficult to restructure representation than loose chunk (Zhang et al., 2019). Problem solvers in tumor-laser radiation problem simulate to use laser power to kill tumor and avoid doing harm to healthy tissue (Duncker, 1945). Xing et al. (2018) used eye movement technique to reveal the impact of embodied guidance on insight by tumor-laser radiation problem. Although spatial and verbal insight problems both emphasize the impasse and representation restructuring as important features of insight, the existing findings revealed differences in the psychological and neural mechanisms of spatial and verbal insight problem (Gilhooly et al., 2011; Cushen and Wiley, 2012). Therefore, it is necessary to explore the impact of WMU ability on spatial insight problem solving and its underlying mechanism, as well as investigate the WMU ability affect which specific phase in the course of spatial insight problem solving.

Previous works investigated the neurocognitive mechanisms of insight problem solving using fMRI (Shen et al., 2016, 2017) and ERP (Zhang et al., 2019) technologies. Eye movement technique, more suitable to investigate the underlying mechanism of spatial insight problem, is also increasingly used (Hegarty and Just, 1993; Grant and Spivey, 2003; Xing et al., 2018). Previous study found that attention is closely related to eye movement (Deubel and Schneider, 1996), and eye gaze is a good indicator of attentional flexibility, which well predicts change in cognitive activity (Kruschke et al., 2005; Rehder and Hoffman, 2005; Blair et al., 2009). Therefore, in the research of spatial insight problem solving, the involving of eye movement technique can help intuitively observe the change of individual’s attention resources in the process of spatial insight problem solving and understand its underlying mechanism.

The present study consists of two experiments. Experiment 1 initially explored the correlation between WMU ability and spatial insight problem solving. Experiment 2 used eye movement technique to directly explore the attention resources change and its underlying mechanism in the process of spatial insight problem solving.

## Experiment 1

### Method

#### Participants

Fifty-seven undergraduate volunteers participated in the experiment (20 males; aged from 17 to 24; mean 18.15 ± 3.1 years), right-handed, having normal or corrected-to-normal vision. All the participants did not encounter the Triangle of Circles or similar problems before, and they signed the informed consent before the experiment and got course credit after. Our sample size was determined using G*power 3.1 (Faul et al., 2009). We assumed that the current study would yield a comparatively large effect size (*d* = 0.8, power = 0.8, *α* = 0.05), in this case the total sample size should be 42 participants, 21 participants, respectively, in one group.

#### Apparatus

The working memory updating task was programmed using the E-prime 2.0 program. The display resolution of the screen is 1,024 × 768. The spatial insight problem was presented on a paper, and participants should draw the answer on a sheet.

#### Procedure

##### Working Memory Updating Task

Matrix updating task designed by Chen and Li (2005) is used as working memory updating task, which could well predict WMU ability (Chen and Li, 2007; Ecker et al., 2010). At first, a 4 × 4 matrix with 16 cells is presented in the center of computer screen. And there are three different color dots (red, yellow, and green), respectively, in one of the cells. The initial locations of these three dots are lasting for 4,000 ms. Afterward, some color arrows (red, yellow, and green) are presented in the center of the matrix successively. These arrows with different directions (left, right, up, or down) are available for 1,500 ms followed by a blank for 500 ms. These color arrows indicated that the dot sharing the same color should move one cell according to the direction of the arrow. There are three series of the number of arrows, respectively, 3, 4, and 5, so arrows of each color are presented once or twice in each trial. The number of arrows varied randomly in each trial, so that participants have no idea about their termination. After all the arrows are presented in one trial, a note is presented on the screen: “Now it’s time to answer.” Only in this time, participants could write three words (red, yellow, and green) to indicate the current locations of dots in 4 × 4 matrix with 16 cells printed on an answer sheet. It should be emphasized that participants were required not to take any notes or keep track of dots using pencil before answering, and they could only update the location of dots mentally. After participants finish answering, they can press the space bar to continue to the next trial. Participants would practice three trials (3, 4, and 5 arrows, respectively) to be familiar with the experiment procedure. In the formal experiment, there are four trials in each series of the number of arrows, 12 trials in total. Each correct answer (correct location of color dot) is rated 1 point, thus 3 points with all correct answers in one trial. The score is recorded from 0 to 36. Getting higher scores means stronger working memory updating ability.

##### Spatial Insight Problem Task

**Figure 1:**
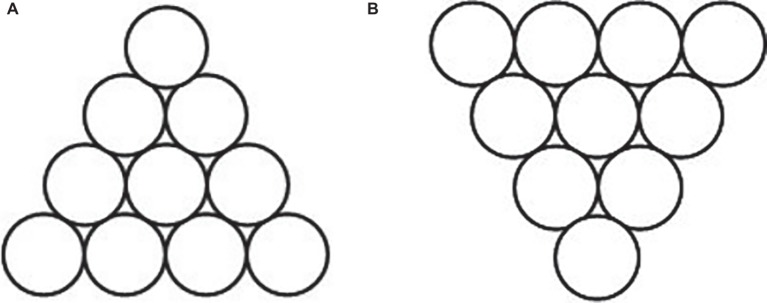
Sample of “Triangle of Circles” problem. Initial state of insight problem **(A)** and target state of insight problem **(B)**.

The “Triangle of Circles” is used to be the materials of the spatial insight problem solving, which is adopted from Cushen and Wiley (2012). As shown in Figure 1A, 10 circles are set up as a perfect triangle pointing toward the top of the page. And the participants are required to move only three circles to reform a perfect triangle pointing toward the bottom of the page, as shown in Figure 1B.

**Figure 2:**
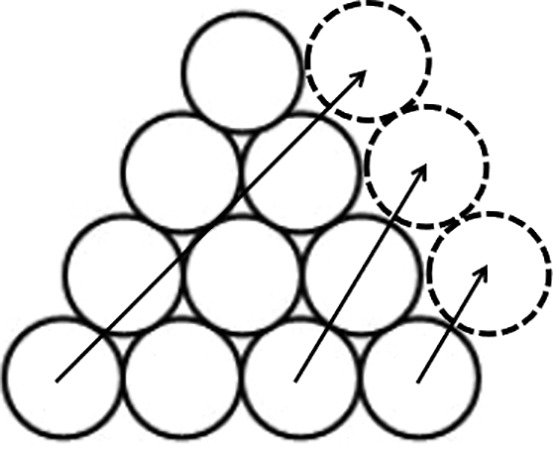
An example of one participant’s answer.

Before the experiment, participants read the requirement of the spatial insight problem, and it is ensured that they understand it. In the formal experiment, participants would be provided 30 s to think and try to figure out the solution. Then, they are given 10 s to draw out three to-be-moved circles and the after-moving positions (Figure 2 shows an example of one participant’s answer). If they do not get the right answer, they would be provided another 30 s to think about the problem and 10 s to answer it. This pattern was repeated as mentioned above until the participants get the right answer or until 10 min running out counting from the first answering.

Two parts should be considered to successfully solve the problem of “Triangle of Circles,” the circles to be moved and the positions after moving. The key circles are the ones on each vertex of the triangle, while the two key positions are on the left and right side of the second row counting from the top, respectively, and the third one is right above the middle of the fourth row. Only find out all right circles and positions can the participants get the correct answer. Each key circle or position is assessed 1 point, with 6 points in total. Each assessment was according to the participants’ answer of to-be-moved circles and after-moving positions. Take one answer shown in Figure 2 as an example, two key circles and one key position are correct, so this answer got 3 points. In the course of problem solving, each answer would be assessed and only the final correct answer got total 6 points.

The order of two tasks was counterbalanced across the participants.

### Results and Analysis

#### Analysis of Time of Spatial Insight Problem Solving

The average score of WMU ability is 24.42 ± 7.02 (M ± SD). Participants with score above the average were divided into high working memory updating ability group (high-level group in short), while participants with score below the average were divided into low working memory updating ability group (low-level group in short; Li et al., 2007; Xing et al., 2017), leaving 28 participants in high-level group and 29 in low-level group.

Forty-one of 57 participants successfully solved the insightful problem, with 21 of them in high-level group and 20 low-level group. The average time of solution for high-level group is 136.71 ± 57.40 (M ± SD) s, while 234.00 ± 151.29 (M ± SD) s for low-level group. Independent sample *T* test is applied to compare the time of solution of both groups. The result showed that the solution time of high-level group is significantly shorter than that of low-level group (*t* = −2.78, *p* = 0.008, Cohen’s *d* = −0.85).

#### Analysis of Representation Pattern Score of Spatial Insight Problem

According to the researches by Cushen and Wiley (2012), each answering assessment can be seen as their problem representation pattern, in order to obtain an online measure of representational change. Therefore, before the participants successfully reached insight (get 6 points), their representational state of concessive answering can be regarded as search or exploration in the faulty problem space. The length of the search process is represented by their solution time. To analyze the restructuring process of insight problem solving, we compared the scores of the participants’ last four answers, which may represent representation pattern.

**Figure 3:**
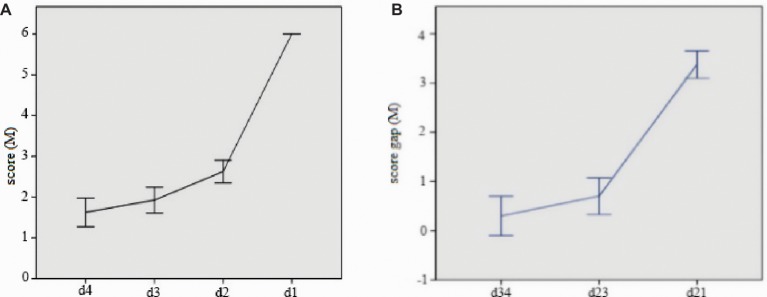
Mean score of last four answers **(A)** and mean score gap between two answers **(B)**. Error bars represent 1 SEM.

For 41 participants who successfully solved the problem, their last four assessments were chosen into the analysis, that is, the forth answer prior to the solution (d4), the third answer prior to the solution (d3), the second answer prior to the solution (d2) and the solution (d1, d1 = 6). Those who solved the problem within four times of answering were excluded in data analysis. As a result, 40 sets of data are effective. The average scores of d4, d3, d2, d1 of these 40 participants are shown in Figure 3A.

A repeated measures ANOVA was conducted on four sets of d score, and *F*(3,117) = 246.49, *p* < 0.001, $\eta_{p}^{2}$ = 0.86. The results showed that there is no significant difference between d3 and d4, while the difference of other sets of scores reached significance. The score gap (represent representational change) of d1–d2 (d12) is significantly more than d2–d3 (d23) or d3–d4 (d34). This means that the gap between final correct representation pattern and the prior one is much larger than the other two periods. The detailed data were shown in Figure 3B.

**Figure 4:**
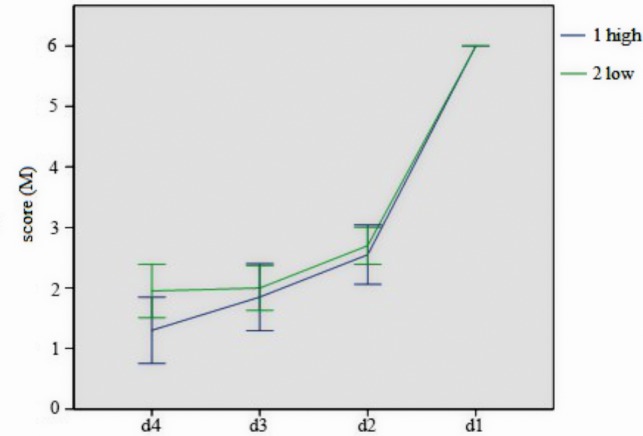
The different mean score of high- and low-level group in last four answers. Error bars represent 1 SEM.

For the analysis of different score of d4, d3, d2, d1 for both high- and low-level group together, it showed that there is no significant difference in d3, d2, and d1 for two groups, with marginal significance in d4, *p* = 0.06. We analyzed mean score gap in high- and low-level group, respectively. For low-level group, there is one difference between d3 and d4, but significant difference existed between the other two comparisons. For high-level group, there is marginal significance between d3 and d4, *p* = 0.08, with significant difference existing between the other two comparisons. The different representation pattern of high- and low-level group was shown in Figure 4.

### Discussion

The solution time of high WMU ability group is significantly shorter than that of low WMU ability group. In other words, the participants in high-level group figured out the solution more quickly. According to three phases of insight problem solving (Ash and Wiley, 2006), it is indicated that participants with high WMU ability would form the correct problem representation more quickly. In the general analysis of representation pattern of insight problem, the comparison of score gap among four sets of d score (assessment of answer representing representation pattern) indicated that there is no significant rise of the solver’s representation level before breaking the impasse (d1). In addition, the following analysis of the score gap of *d* score showed that the score gap of d12 (d1–d2) is significantly larger than that of d23 (d2–d3) or d34 (d3–d4). Therefore, it can be inferred that the process between the impasse (d4, d3, d2) and the impasse overcoming (d1, representation restructuring phase) is not an incremental and gradual one but a sudden and immediate one. Furthermore, WMU ability has no influence on the tendency of this sudden and immediate process in spatial insight problem solving. In other words, this process involves little working memory, which further indicated that the process of overcoming the impasse and representation restructuring is tended to be spontaneous.

According to the analysis of representation pattern in insightful problem for both high- and low-level groups, there is no difference for the score in d1, d2, and d3. However, there is marginal difference in score of representation pattern in d4 (early problem space search phase). And the representation pattern of low-level group is similar to that of the general group, but there is significant difference between each two of four sets of scores (one marginally significant). Therefore, in the problem space search phase, participants with low WMU ability are inclined to adopt analytical search mode, while participants with high WMU ability tended to adopt divergent search mode. Hence, in the early phase of problem space search, the problem representation of participants with high WMU ability is far away from the target state but they can reach the target state quickly.

We believe that the influence of individual cognitive resources on insight problem space search mode is more reflected in the distribution of resources and the cooperation between different functions. That is, the divergent resource allocation of high-level groups enables attention and cognitive resources to better update and restructure the problem representation, then to search for the correct problem representation. Eye movement technique can intuitively observe the change of attention recourses, so we used it to directly explore the attention resource change and to underlie the mechanism in the process of spatial insight problem solving in Experiment 2.

## Experiment 2

### Method

#### Participants

Sixty-two undergraduate volunteers participated in the experiment (25 males; aged from 18 to 26; mean 20.15 ± 2.51 years), right-handed, having normal or corrected-to-normal vision. All the participants did not encounter Triangle of Circles or similar problem before, and they signed the informed consent before the experiment and got course credit after.

#### Apparatus

The working memory updating task and spatial insight problem task were programmed using the E-prime 2.0 program. The display resolution of the screen is 1,024 × 768. Eye movement data were recorded by EYELINK II with a sampling rate of 250 HZ. The movement of right eye was recorded.

#### Procedure

**Figure 5:**
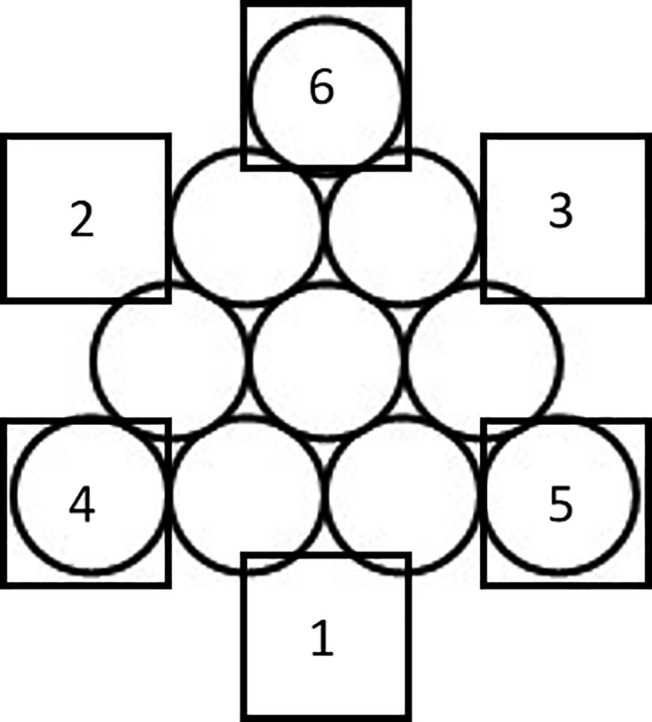
The distribution of AOIs.

The procedure of working memory updating task is the same as Experiment 1. The procedure of spatial insight problem task is similar to Experiment 1 with the difference that the insight problem in Experiment 1 is presented by paper and answered by drawing, while the problem in Experiment 2 is presented by computer screen and answered verbally. In order to ensure that participants could consider the insight problem continuously, if they do not come up with an answer during the answering phase, there is no need to answer. On the other hand, if the insight happens during the observation phase, participants can immediately press the space bar to enter the answering phase, during which they could answer the question verbally. Eye movements in both two phases were recorded. We set the position of the three key circles and the target position as the Area of Interest (AOI), which is represented by square boxes. The AOI 4, 5, and 6 are key circles, and the AOI 1, 2, and 3 are key positions, as shown in Figure 5.

### Results and Analysis

#### Analysis of Time of Spatial Insight Problem Solving

The data of 11 participants who did not successfully answer the spatial insight problem were excluded, remaining 51 subjects in the analysis. For WMU ability, the participants above the average score 21.96 ± 6.87 (M ± SD) were rated as group with high WMU ability (high-level group for short), while the participants below the average score were rated as group with low WMU ability (low-level group for short), leaving 26 participants in the high-level group and 25 in the low-level group.

The average solution time of the high-level group was 150.77 ± 61.25 (M ± SD) s, and the average solution time of the low-level group was 232.00 ± 114.31 (M ± SD) s. Independent sample *T* test was conducted on the time of spatial insight problem between high- and low-level groups. The results showed that the problem-solving time of high-level group was significantly shorter than that of low-level group, *t*(49) = −3.18, *p* = 0.003, Cohen’s *d* = −0.88.

#### Analysis of Eye Movement Data

Thirty-second observation phase and 10-s answering phase are together regarded as one block. Since the number of blocks required to solve the spatial insight problem in the high-level group was 3.78 ± 1.53 (M ± SD) blocks, the eye movement data in last three blocks of each participant (three blocks count downward from the last block) were chosen for analysis. To help clarity, the last block was the block in which participants successfully solve the problem, and the data in the last block was also included into analysis. Nine participants in the high-level group completed the spatial insight problem task within two blocks, so these data were excluded from final analysis, leaving 25 participants in low-level group while 17 in high-level group. We set Block 3 referring to the last but two blocks, Block 2 referring to the last but one block, Block 1 referring to the last block in which participants successfully solved the spatial insight problem.

Fixation refers to eyes keeping comparatively static during eye movement, and the number of fixations could effectively reflect the processing cognitive load of the certain stimuli, with larger cognitive load accompanying with larger number of fixations (Yan et al., 2013). Owing that AOIs we settled only occupied some parts of the Triangle of Circles, fixation ratio in AOIs was selected as an indicator of eye movement, which refers to the proportion of the number of fixations in AOIs and the number of fixations falling on the entire stimulus. A 2 (WMU ability) × 3 (block) repeated measures ANOVA was conducted on the fixation ration in AOIs, with WMU ability being between-subject variable and block within-subject variable. The results showed that the main effect of block was significant, *F*(2,80) = 7.62, *p* = 0.001, $\eta_{p}^{2}$ = 0.16, but the main effect of WMU ability was not significant, *F*(1,40) = 0.046, *p* = 0.83, $\eta_{p}^{2}$ = 0.001. There is no significant interaction, *F*(2,80) = 0.052, *p* = 0.95, $\eta_{p}^{2}$ = 0.001. Further analysis found that the difference between Block 3 and Block 1 was significant, *p* = 0.004; the difference between Block 2 and Block 1 was significant, *p* < 0.001; the difference between Block 3 and Block 2 is not significant, *p* = 0.50.

**Figure 6:**
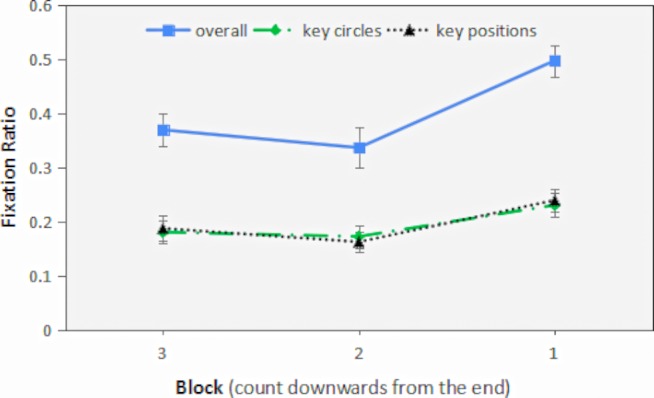
The fixation ration in AOIs in last three blocks. Error bars represent 1 SEM.

The information in AOIs is critical to spatial insight problem solving. Among them, the AOIs 4, 5, and 6 are key circles, and the AOIs 1, 2, and 3 are key positions. To verify the successful solution of spatial insight problem depends which types of information, a 2 (type of AOIs) × 3 (block) repeated measures ANOVA was conducted on the fixation ration, with type of AOIs and block being within-subject variables. The results showed that the main effect of block is marginally significant, *F*(2,82) = 2.77, *p* = 0.068, $\eta_{p}^{2}$ = 0.063, but the main effect of type of AOIs is not significant, *F*(1,41) = 0.225, *p* = 0.64, $\eta_{p}^{2}$ = 0.005. The interaction was either not significant, *F*(2,82) = 1.38, *p* = 0.26, $\eta_{p}^{2}$ = 0.032. Further analysis found that the difference between Block 3 and Block 1 was significant, *p* = 0.03; the difference between Block 2 and Block 1 was significant, *p* = 0.019; the difference between Block 3 and Block 2 is not significant, *p* = 0.61. See Table 1 and Figure 6 for the detailed fixation ratio in each block.

**Table 1:** Descriptive statistics of fixation ratio in AOIs (M ± SD).

AOIs	Block 3	Block 2	Block 1
Overall	0.37 ± 0.03	0.33 ± 0.03	0.50 ± 0.03
Key circles	0.18 ± 0.02	0.17 ± 0.02	0.23 ± 0.02
Key positions	0.19 ± 0.02	0.16 ± 0.02	0.24 ± 0.02

Note: Block 3 refers to the last but two blocks; Block 2 refers to the last but one block; Block 1 refers to the last block.

### Discussion

The last block in which participants successfully solve the spatial insight problem is regarded as insight phase, while other blocks are regarded as non-insight phase. In the process of spatial insight problem solving, the fixation ration in AOIs of insight phase is significantly higher than that of non-insight phase, indicating that more attention resources are allocated to the key circles and key positions when the participants reached insight. Moreover, there is no significant difference between the fixation ration of key circles and key positions, indicating that it is necessary to focus on these two key pieces of information instead of one single piece of information to achieve insight. In the previous problem space search phase, the participants did not pay enough attention to the key circles and key positions, or just focused on some parts of them, so that the participants form the incorrect problem representation and were stuck in impasse. The eye movement reflects the change of attentional and cognitive recourses to a certain extent (Godijn and Pratt, 2002). When the participants attempted to solve the spatial insight problem, they would try to move the circles to other positions. In the process, the participants needed to remember the previous-moving circles, the after-moving positions, and the reconstructed shape of the stimulus, after that participants should judge whether the new shape of circles is the perfect triangle pointing to the bottom. Each step in this process is closely related to the WMU ability. Participants with high WMU ability may remember the previously wrong representation of information, so they found key information and reached insight faster than those with low WMU ability. They may repeat the same misrepresentation of the problem, leading to spend longer time to solve the spatial insight problem.

From the whole course of spatial insight problem solving, the fixation ration in AOIs of the last block is significantly higher than that of its previous block, while there is no significant difference in the fixation ratio in AOIs between the last but two blocks and the last but one block. The changing pattern of the fixation ration in AOIs in Experiment 2 is similar to that of representation pattern in Experiment 1. The results showed that the underlying mechanism of insight problem solving is sudden rather than incremental, although there is difference in the length of time to solve the insight problem, which provides some evidence for that representation restructuring phase is a spontaneous process.

## General Discussion

The current study is an expansion and supplement of Cushen and Wiley (2012) to make it clearer to understand the psychological process of insight problem solving. First, Cushen and Wiley (2012) defined the participants’ problem representation by rating the importance of each problem item. And in current experiment, the participants were not required to rate the circles but to directly offer their immediate answers, which represent more specific representation. Second, the time limit for the participants to think is reduced from 1 min to 30 s, which makes it clearer to understand the process of the problem representation restructuring. Third, even if the participants found out the key circles, they might still fail to solve the problem for not getting the key positions. Therefore, scores are given according to both key circles and key positions in Experiment 1, and AOIs are settled for both key circles and key positions in Experiment 2. Finally, to investigate the problem representation, participants were asked to draw the answer on the answer sheet in Experiment 1 every 30 s, which may interfere with their thinking course. It is different from the solution to the insight problem in real life, which should be smooth and continuous. So, Experiment 2 improved the design to avoid this interfering influence.

Quantities of studies were conducted to explore whether the underlying mechanism of insight problem solving is conscious, cognitive recourses needed or spontaneous, automatic, and implicit. Murphy (2005) investigated the correlation between the insight problem solving task and the graphical fluency task (measurement of the inhibition of dominant responses and the transformation of the basic response) and found that there is inhibition and transformation involving in the solution of the insight problem.

However, some researchers indicated that restructuring is an all-or-none and unreportable problem-solving process in insight problem solving (Metcalfe and Wiebe, 1987; Ash and Wiley, 2006; Ash et al., 2009). Fleck (2008) compared four insight and non-insight problems (analytic problems) and found the correlation between working memory span and non-insight problem solving, as well as the correlation between insight problem solving and short-term information storage (without attentional control), which supports the spontaneous theory of restructuring in insight. It also revealed that in insight problem solving, control processing accounts for a small proportion and automatic processing accounts for a large proportion. Lavric and Forstmeier (2000) also drew similar conclusions that dual processing tasks (simultaneous computing tasks) having less interference with insight problem solving. Ash and Wiley (2006) compared the different size of the faulty initial search space and found that higher capacity to control attention help navigate the initial space. This result also explains the effect of automatic processing on restructuring for that small search spaces only need to be restructured, while large search spaces need to be searched and restructured. Shanker (1995) proposed that unconsciousness does not have a rigorous filter like consciousness. In the course of solving creative problems, problem solvers unconsciously pick out clues irrelevant or new to the problem and link them, which explain the facilitate role of unconscious thinking in insight problem solving to some extent.

The Representational-Change Theory (Ohlsson, 1992; Knoblich et al., 1999) asserts that insight only occurs when the problem solvers stuck in impasse, which is the result of the current problem representation acts as the memory predictor of the incorrect initial problem representation. Individuals only change the current problem representation and form a new memory predictor, then extract the relevant information from the memory can overcome the impasse, and then partial or complete insight may occur. Later, Knoblich et al. (2001) perfected this theory, arguing that the initial representation of problems established by problem solvers enables unimportant knowledge be activated, creating obstacles to problem solving. Only by restructuring the representation of this problem and changing the state of knowledge activation, they can solve the insight problem successfully. And Knoblich et al. (2001) also pointed out that representation restructuring mainly relies on two mechanisms, constraint relaxation and chunk decomposition. Among them, the entire constraint is the most difficult to relax, because in this case, it is necessary to transform the representation of the whole problem; and the partial restriction is much easier, because the partial constraint only affects part of the problem representation. The same is true for chunk decomposition, which is easier to break down for loose chunks than tight chunks (Zhang et al., 2019). Knoblich et al. (2001) also verified the theoretical hypothesis of constraint relaxation and chunk decomposition by solving the matchstick arithmetic problem. After problem solvers see the stimulus and get the negative feedback in the insight condition, they need to break his previous guessing rules and generate new connection between the answer and the puzzle as well as correct representation, in which process more attention recourses and working memory are needed. Individuals with low WMU ability have formed an incorrect problem representation, so they spend more time in impasse than individuals with high WMU ability.

Analysis of representation pattern in Experiment 1 found that search mode of individuals with low WMU ability in problem search space is similar to that in analytical problem solving, which is analytical search mode, while individuals with high WMU ability adopt the divergent search mode. Compared with analytical search mode, the divergent search mode enjoys higher requirements in terms of cognitive resources, state of consciousness, attention span, and divergent thinking. WMU ability mainly shows the individual’s working memory capacity and information updating ability (Collette and Linden, 2002) and involves the participation of cognitive resources and conscious process. The negative correlation between WMU ability and the time of spatial insight problem solving in both Experiments 1 and 2 showed that WMU ability plays an important role in the problem space search phase. It is believed that there are quantities of cognitive resources participating and investing in the initial representation phase and problem space search phase in the course of spatial insight problem solving.

In addition, Experiment 1 found significant difference of representation pattern between representation restructuring phase and impasse; Experiment 2 found significant difference of fixation ratio in AOIs between insight phase and non-insight phase. Both two experiments proved that the restructuring process of insight is sudden and immediate. The representation restructuring phase is not affected by the ability of working memory updating, which leads to that the representation restructuring may be a spontaneous processing.

In conclusion, the current study found that insight basically conforms to the Representational-Change Theory. According to the interpret of different phases in the course of insight problem solving (Ash and Wiley, 2006), the previous two phases (initial problem representation phase and problem space search phase) are more involved of conscious processing, and the last phase (representation restructuring process) is more inclined to be a spontaneous process. The current study also provided some evidence for that the solution of insight problem is a dual thinking processing.

There are several limitations in this research, which could be further improved and investigated in the future. (1) The test of the individual’s WMU ability only used Matrix Updating task, and the evaluation and prediction of the WMU ability can be conducted in a more integrated and comprehensive method (Ecker et al., 2010; von Bastian et al., 2015). Furthermore, the division of high and low WMU ability group could be upper and lower quarter or third (Gelman and Park, 2009), to make the comparison more precisely. (2) The current study chose classic spatial insight problem, but previous studies have found that the size of problem space would influence insight problem solving (Ash and Wiley, 2006), so we consider further investigating the effect of WMU ability on different size of problem space. (3) The exact moment of insight is difficult to define. Some conservative participants may rethink the problem again after insight to verify their answer. In future, we can combine eye movement and ERPs technology to explore the solution of insight in a more precise time course.

## Conclusion

1. The ability of working memory updating affects the solution of spatial insight problem solving, and individuals with high WMU ability spend significant shorter time on spatial problem solving than individuals with low WMU ability. The influence occurs in the problem space search phase.
2. The representational score and fixation ratio in AOIs of insight phase is significantly higher than that of non-insight phase, indicating the representation restructuring phase of spatial insight is a sudden and spontaneous process and is not affected by the ability of WMU.
3. There is no significant difference in the fixation ratio in AOIs of the key circles and key positions, indicating that the solution to the spatial insight problem solving needs to pay attention to both types of key information.

## Ethics Statement

The study reported in the manuscript entitled “The Effect of Working Memory Updating Ability on Spatial Insight Problem Solving: Evidence from Behavior and Eye Movement Studies” has been approved by the Institutional Review Board at Guangzhou University.

## Author Contributions

QX designed the study, ZL and JH assisted with data collection. All authors wrote the manuscript and analyzed and interpreted the data.

### Conflict of Interest Statement

The authors declare that the research was conducted in the absence of any commercial or financial relationships that could be construed as a potential conflict of interest.
